# Incidental Diagnosis of a Well-Differentiated Appendiceal Neuroendocrine Tumor Following Appendectomy for Complicated Acute Appendicitis: A Case Report

**DOI:** 10.7759/cureus.110421

**Published:** 2026-06-07

**Authors:** Brayan Benguechea, Oscar A Castro, Andrea N Garza Cisneros, Daniel A Martinez Espinal, Irasema Salazar-Marquez

**Affiliations:** 1 General Surgery, Instituto Mexicano del Seguro Social, Ciudad Juárez, MEX; 2 General Surgery, Hospital Regional Monterrey Instituto de Seguridad y Servicios Sociales de los Trabajadores del Estado (ISSSTE), Monterrey, MEX; 3 Surgery, Instituto Mexicano del Seguro Social, Ciudad Juárez, MEX

**Keywords:** acute appendicitis, appendiceal neuroendocrine tumor, neuroendocrine neoplasm, neuroendocrine tumor, open appendicectomy

## Abstract

Appendiceal neuroendocrine tumors are the most common primary neoplasms of the appendix and are frequently diagnosed incidentally following appendectomy performed for suspected acute appendicitis. We present a case of a 72-year-old woman with a history of hypertension and hypothyroidism who presented with a 24-h history of right lower quadrant abdominal pain associated with nausea and low-grade fever. Non-contrast abdominal computed tomography demonstrated findings consistent with acute appendicitis. An urgent open appendectomy was performed, and intraoperative findings revealed complicated appendicitis with pelvic inflammatory fluid, fibrinopurulent exudates, and distal appendiceal necrosis. Histopathological examination demonstrated a well-differentiated appendiceal neuroendocrine tumor (NET G1, WHO grade 1), with negative surgical margins, absence of lymphovascular invasion, and a mitotic index of less than 1/mm², associated with acute suppurative appendicitis. Given the favorable histopathological findings, no complementary right hemicolectomy was performed, and the patient was managed with clinical follow-up. The postoperative course was uneventful, with oral intake initiated on postoperative day one, diet advancement on day two, and hospital discharge on day three. This case highlights the importance of routine histopathological examination of all appendectomy specimens and reviews current considerations for risk stratification and therapeutic decision-making in patients with appendiceal neuroendocrine tumors.

## Introduction

Appendiceal neuroendocrine tumors are uncommon neoplasms that are most often diagnosed as incidental findings following appendectomy performed for suspected acute appendicitis. They arise from enterochromaffin cells of the diffuse neuroendocrine system and account for approximately 50-70% of all appendiceal neoplasms, representing the most common primary neoplasm of the appendix [1,2]. These tumors are typically located at the appendiceal tip and are generally characterized by indolent biological behavior with an excellent long-term prognosis. Because the majority of patients present with clinical manifestations indistinguishable from an inflammatory appendiceal process, preoperative diagnosis is rare and is usually established only after histopathological examination of the surgical specimen [2,3].

Although these tumors are generally well-differentiated and associated with favorable biological behavior, the identification of specific histopathological features may significantly influence subsequent management. Factors such as tumor size, mesoappendiceal invasion, lymphovascular invasion, proliferative activity, tumor grade, and surgical margin status are critical for assessing the risk of residual or metastatic disease and determining the need for additional treatment. Current management recommendations and decisions regarding completion of right hemicolectomy are largely based on these pathological characteristics [1-3].

We present a case of a 72-year-old woman with complicated acute appendicitis in whom histopathological examination incidentally revealed a well-differentiated appendiceal neuroendocrine tumor, highlighting the importance of individualized therapeutic decision-making and postoperative follow-up.

## Case presentation

A 72-year-old woman with a medical history of hypertension and hypothyroidism presented to the emergency department. She reported a 24-h history of sudden-onset abdominal pain localized to the right lower quadrant. The pain was colicky in nature, rated 9/10 on the visual analog scale, and worsened with movement and abdominal palpation. Associated symptoms included nausea and low-grade fever. She denied vomiting, changes in bowel habits, or urinary symptoms.

On physical examination, the patient was awake, alert, and oriented, with stable vital signs. Abdominal examination revealed a soft and depressible abdomen with tenderness to deep palpation in the right lower quadrant. Localized signs of peritoneal irritation were present, including positive McBurney, Rovsing, Dunphy, psoas, and rebound signs. Laboratory studies obtained on admission are summarized in Table 1.

**Table 1:** Preoperative laboratory findings. PT: prothrombin time; aPTT: activated partial thromboplastin time; INR: international normalized ratio

Parameters	Results	Reference range
PT	12.1 s	11-13.5 s
aPTT	28 s	25-30 s
INR	0.8 s	0.8-1.2 s
Hemoglobin	12.6 g/dL	12-16 g/dL
Platelet count	186 × 10³/µL	150-450 × 10³/µL
Leukocytes	6.4 × 10³/µL	4.0-10.0 × 10³/µL
Neutrophils	67%	40-75%
Lymphocytes	25%	20-45%
Glucose	94 mg/dL	70-100 mg/dL
Urea	33.4 mg/dL	15-40 mg/dL
Creatinine	0.6 mg/dL	0.5-1.1 mg/dL

Non-contrast abdominal computed tomography demonstrated inflammatory changes in the right lower quadrant consistent with acute appendicitis (Figure 1). Based on the clinical and radiological findings, an urgent open appendectomy was performed using the Pouchet technique. Intraoperatively, inflammatory fluid was identified in the pelvic cavity and right lower quadrant. The appendix was located in a pelvic position and measured approximately 9 cm in length. The appendiceal base appeared grossly preserved. The resected specimen showed a thickened and inflamed appendix with distal enlargement (Figure 2).

**Figure 1:**
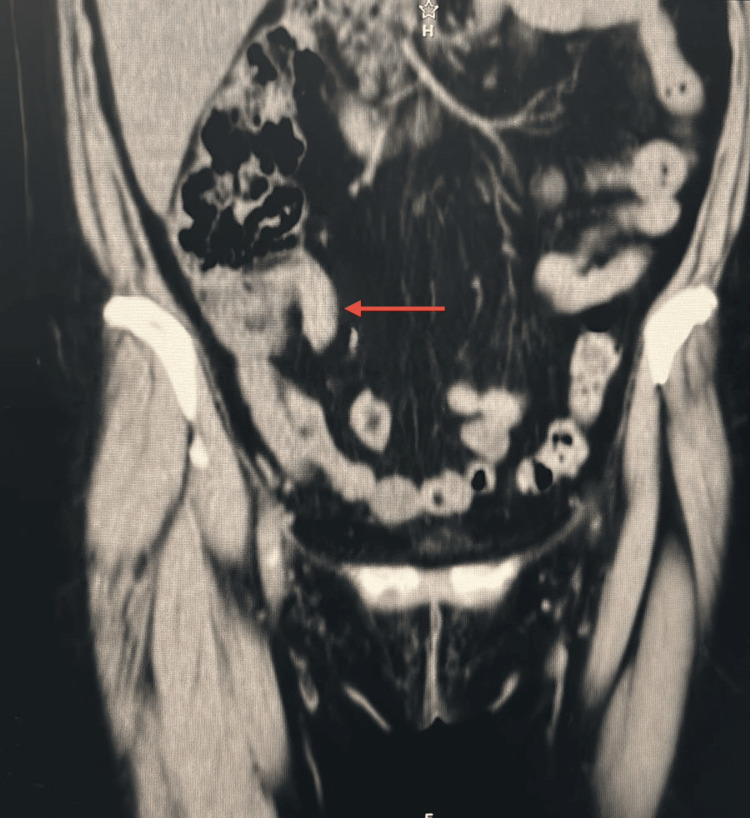
Non-contrast abdominal computed tomography. Coronal non-contrast abdominal CT image showing focal inflammatory changes in the right lower quadrant (red arrow), findings consistent with acute appendicitis.

**Figure 2:**
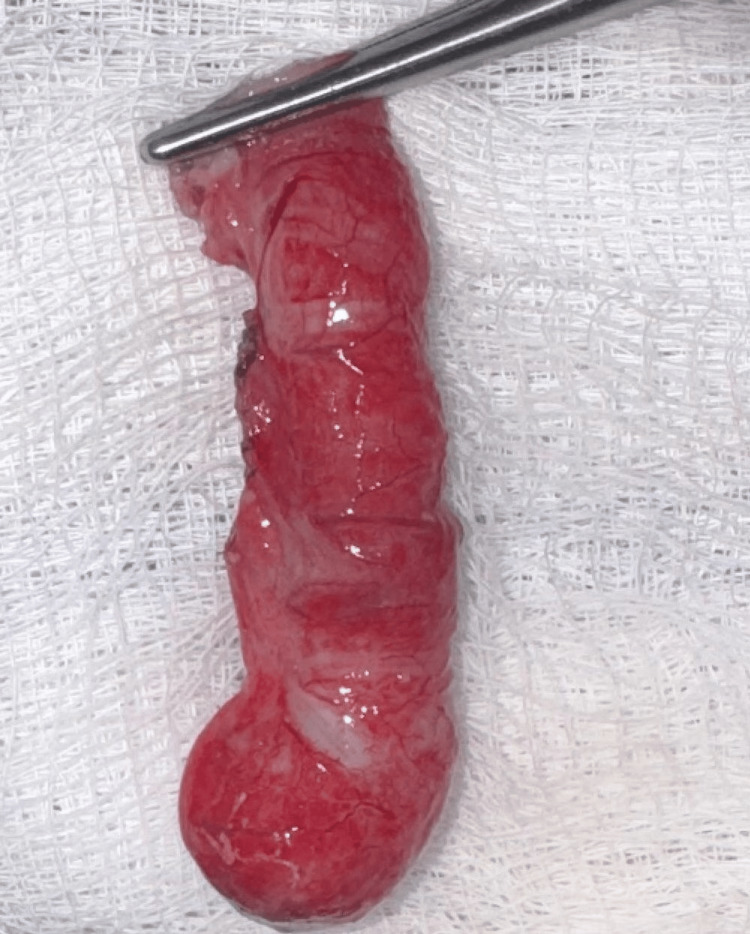
Resected appendiceal specimen obtained during open appendectomy. The image showing a thickened and inflamed appendix with distal enlargement.

Histopathological examination described a cecal appendix measuring 9 × 1.3 cm with an opaque serosal surface. On sectioning, a dilated lumen containing fecal material and a thickened wall were observed. Microscopic analysis revealed a well-differentiated neuroendocrine tumor (NET G1, WHO grade 1) with diffuse infiltration of the appendiceal wall. No lymphovascular invasion was identified, and the mitotic activity was less than 1 mitosis/mm². Surgical margins were free of tumor involvement. Additionally, acute suppurative appendicitis with inflammatory extension to the surgical margin corresponding to the appendiceal base was reported, without evidence of tumor involvement of the margin.

Microscopic examination demonstrated a neoplastic proliferation arranged in nests and islands of monomorphic cells with round nuclei and finely granular “salt-and-pepper” chromatin, findings consistent with a well-differentiated neuroendocrine tumor (Figure 3).

**Figure 3:**
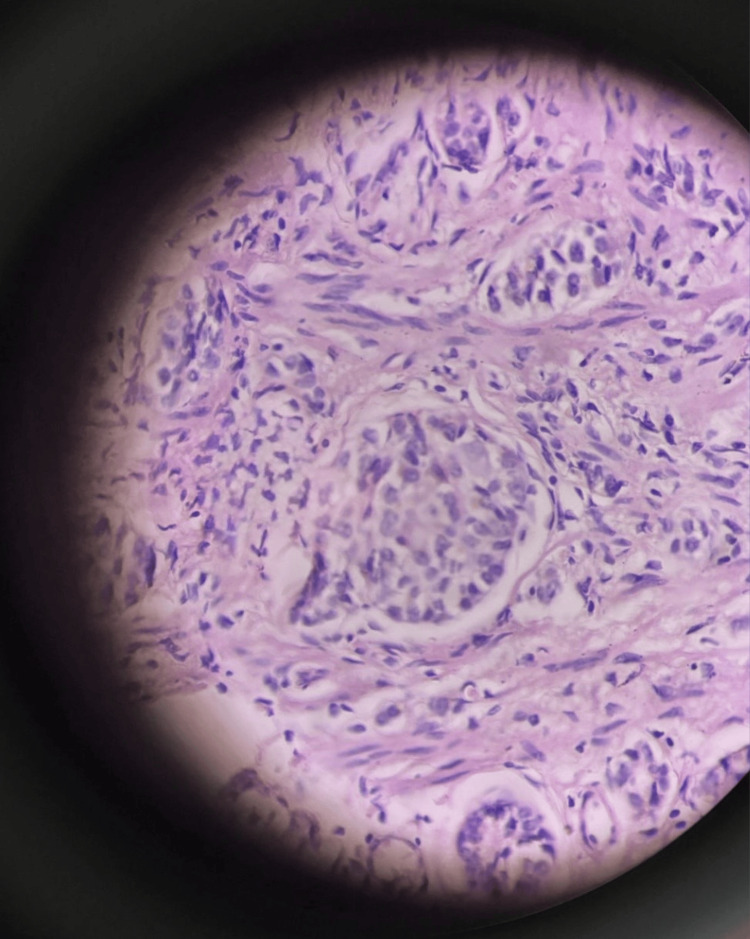
Histopathological findings of a well-differentiated appendiceal neuroendocrine tumor. Hematoxylin and eosin-stained section showing nests and islands of uniform neuroendocrine cells with characteristic finely granular “salt-and-pepper” chromatin, consistent with a well-differentiated appendiceal neuroendocrine tumor (NET G1, WHO grade 1).

Following review of the histopathological findings, no complementary right hemicolectomy was indicated. Considering the negative surgical margins, absence of lymphovascular invasion, and low mitotic index, the patient was managed with outpatient clinical follow-up.

The postoperative course was uneventful. Oral intake was initiated on postoperative day one without nausea, vomiting, or other signs of gastrointestinal intolerance. On postoperative day two, the diet was advanced appropriately, and the patient remained hemodynamically stable with no evidence of infectious or surgical complications. After a three-day hospital stay and a favorable clinical recovery, she was discharged with an outpatient follow-up appointment scheduled.

## Discussion

Appendiceal neuroendocrine tumors (aNETs) are the most common primary neoplasms of the appendix and are frequently identified as incidental findings following appendectomy performed for suspected acute appendicitis [1-3]. Despite their relative rarity, they account for approximately 50-70% of all appendiceal neoplasms and are generally associated with favorable long-term outcomes when diagnosed at an early stage [1-3].

In most patients, the initial clinical presentation is determined by the inflammatory appendiceal process rather than the underlying neoplasm, making preoperative diagnosis difficult. This presentation is thought to result from partial luminal obstruction caused by the tumor, leading to increased intraluminal pressure, bacterial overgrowth, vascular compromise, and secondary inflammation, ultimately producing symptoms indistinguishable from acute appendicitis [1-3]. In the present case, the patient presented clinical and radiological findings consistent with complicated acute appendicitis, without any evidence suggesting an appendiceal neoplasm prior to surgical intervention.

The definitive diagnosis was established through histopathological examination of the surgical specimen, reinforcing the importance of routinely submitting all appendectomy specimens for pathological evaluation. Although most appendiceal neuroendocrine tumors exhibit indolent biological behavior, their identification carries important prognostic and therapeutic implications, particularly when features associated with a higher risk of residual or metastatic disease are present [1-3].

From a histopathological perspective, most appendiceal neuroendocrine tumors are well-differentiated, low-grade neoplasms characterized by favorable biological behavior and a low likelihood of recurrence or metastasis [1-3]. In our patient, histopathological examination demonstrated a well-differentiated neuroendocrine tumor (NET G1, WHO grade 1), with negative surgical margins, absence of lymphovascular invasion, and a mitotic index of less than 1 mitosis/mm². These findings are considered favorable prognostic factors and are associated with a low risk of recurrence or metastatic spread [1,3]. Furthermore, the observed microscopic pattern, characterized by nests and islands of monomorphic cells with finely granular “salt-and-pepper” chromatin, corresponds to the classic morphology described for well-differentiated appendiceal neuroendocrine tumors [1]. A limitation of this report is the absence of immunohistochemical studies, as the diagnosis was established based on the characteristic histopathological findings reported by the Department of Pathology.

One of the most important aspects in the management of appendiceal neuroendocrine tumors is determining whether appendectomy alone is sufficient treatment or whether completion right hemicolectomy is indicated. Current recommendations emphasize the need for individualized therapeutic decision-making based on histopathological variables, such as tumor size, tumor location within the appendix, mesoappendiceal invasion, lymphovascular invasion, proliferative index, and surgical margin status [1-3]. Although right hemicolectomy has traditionally been recommended for tumors larger than 2 cm or those with high-risk features, recent evidence has supported a more conservative approach in selected patients with completely resected, well-differentiated tumors [1-3].

In our patient, a complete right hemicolectomy was not performed. This decision was based on the presence of negative surgical margins, absence of lymphovascular invasion, and a mitotic index of less than 1 mitosis/mm², findings that suggest favorable biological behavior [1-3]. Although information on tumor size, Ki-67 index, and depth of mesoappendiceal invasion was unavailable, there were no histopathological findings suggestive of high-risk disease. Therefore, clinical follow-up without additional surgical intervention was considered an appropriate management strategy [1-3].

Another relevant aspect of this case was the coexistence of acute suppurative appendicitis with inflammatory extension to the appendiceal base. It is important to distinguish between inflammatory involvement of the surgical margin and tumor involvement of the margin, as these scenarios have different clinical implications. Tumor involvement of the surgical margin may indicate residual disease and can influence the indication for completion right hemicolectomy or closer oncological surveillance, whereas inflammatory extension to the margin reflects the severity of the acute inflammatory process and does not, by itself, imply residual neoplastic disease [1-3]. In our patient, although the inflammatory process extended to the appendiceal base, histopathological examination confirmed tumor-free surgical margins, eliminating the need for additional procedures based solely on margin interpretation [1-3].

The main limitations of this case include the absence of immunohistochemical characterization and the lack of histopathological variables currently considered essential for risk stratification, particularly tumor size, Ki-67 index, and depth of mesoappendiceal invasion. Although the diagnosis was established based on the characteristic morphologic features of a well-differentiated neuroendocrine tumor, immunohistochemical markers such as chromogranin A, synaptophysin, and Ki-67 could have provided additional diagnostic and prognostic information. Availability of these parameters would have allowed a more precise assessment of the indication for additional surgery and a more comprehensive comparison with contemporary international guideline recommendations [1-3]. Additionally, high-resolution histopathological images and documented magnification levels were not available for review, limiting the ability to provide a more detailed pathological illustration of the lesion.

Our findings are consistent with other case reports in the literature in which appendiceal neuroendocrine tumors were identified incidentally following appendectomy performed for suspected acute appendicitis [4,5]. Carballo-Torres et al. described a similar clinical scenario, emphasizing that the definitive diagnosis was established only through histopathological examination of the surgical specimen, reinforcing the importance of routine pathological evaluation of all appendectomy specimens regardless of clinical or intraoperative findings [5].

Recent literature suggests that most incidentally diagnosed appendiceal neuroendocrine tumors have an excellent prognosis and can be adequately managed with appendectomy alone [1-3]. Nevertheless, cases with regional lymph node involvement or more aggressive histopathological characteristics have been reported and require individualized management strategies [4].

A noteworthy aspect of our case is the patient's age at presentation. Although appendiceal neuroendocrine tumors may occur at any age, most series report a higher incidence in younger and middle-aged patients [1,2]. The diagnosis of this neoplasm in a 72-year-old woman highlights that, although uncommon, this entity can occur in elderly patients and should be considered among potential incidental findings in any appendectomy specimen. Similarly, recent reports have demonstrated that these neoplasms may be identified across different age groups and clinical settings, including pediatric patients, illustrating the broad variability in their clinical presentation [6].

Finally, this case emphasizes the importance of routine histopathological examination of all appendectomy specimens, even in the setting of complicated acute appendicitis. The identification of an incidental neuroendocrine tumor may influence follow-up strategies, prompt multidisciplinary evaluation, and affect subsequent therapeutic decisions that would not have been considered based solely on clinical or intraoperative findings. Furthermore, this case highlights the importance of comprehensive pathology reporting. Although tumor size, Ki-67 index, and mesoappendiceal invasion were not available in the present case, these parameters are recognized as essential components of contemporary risk stratification systems and play a key role in therapeutic decision-making and follow-up planning for patients with appendiceal neuroendocrine tumors [1-3]. Their absence limited a more precise assessment of the patient's risk profile and represents an important limitation of this report.

## Conclusions

Appendiceal neuroendocrine tumors are uncommon neoplasms that are often identified as incidental findings following appendectomy performed for acute appendicitis. This case highlights the importance of routine histopathological examination of all appendectomy specimens, as clinically unsuspected neoplasms may be detected even in patients presenting with complicated acute appendicitis. In our patient, the presence of a well-differentiated neuroendocrine tumor (NET G1, WHO grade 1), negative surgical margins, absence of lymphovascular invasion, and a low mitotic index supported management with appendectomy alone and clinical follow-up. Comprehensive histopathological evaluation remains essential for appropriate risk stratification and individualized therapeutic decision-making in patients with appendiceal neuroendocrine tumors.
